# Sustainability of Hiking in Combination with Coaching in Cardiorespiratory Fitness and Quality of Life

**DOI:** 10.3390/ijerph19073848

**Published:** 2022-03-24

**Authors:** Daniela Huber, Michaela Mayr, Arnulf Hartl, Sandra Sittenthaler, Eva Traut-Mattausch, Renate Weisböck-Erdheim, Johanna Freidl

**Affiliations:** 1 Institute of Ecomedicine, Paracelsus Medical University Salzburg, Strubergasse 21, 5020 Salzburg, Austria; daniela.huber@pmu.ac.at (D.H.); michaela.mayr@pmu.ac.at (M.M.); renate.erdheim@pmu.ac.at (R.W.-E.); johanna.freidl@pmu.ac.at (J.F.); 2 Zentrum für Kinder und Jugendliche e.V., 84503 Altoetting, Germany; sandra.sittenthaler@googlemail.com; 3 Department of Psychology, University of Salzburg, 5020 Salzburg, Austria; eva.traut-mattausch@plus.ac.at

**Keywords:** green exercise, coaching, quality of life, cardiorespiratory fitness, physical activity

## Abstract

Although strong evidence shows that physical inactivity and sedentary behavior are associated with many negative health outcomes, inactive lifestyles are still increasing. Consequently, new approaches must be developed to increase adherence to an active lifestyle and hence a longer life. Green exercise and health coaching could be effective ways to induce long-lasting lifestyle changes geared towards more physical activity. In this randomized controlled trial, we investigated the effects of mountain hiking and psychological coaching on adults with a sedentary lifestyle. The coaching group (*n* = 26) participated in a 7-day guided hiking program with three personal coaching sessions, whereas the hiking group (*n* = 32) received no coaching. The effects on aerobic capacity, spirometry and quality of life were assessed at baseline (day 0), after the intervention week (day 7) and after 80 days. Fully nonparametric statistical analysis revealed a gender-based effect for aerobic capacity—the female participants of the coaching group showed a greater improvement (*p* = 0.03) than the hiking group. No significant effects were found for spirometry. Quality of life parameters improved in both groups. In conclusion, both green exercise and health coaching are capable of inducing improvements in health-related quality of life and cardiorespiratory fitness. No superior effects of health coaching were found.

## 1. Introduction

Although strong evidence shows that physical inactivity and sedentary behavior are associated with many adverse health effects, the “global pandemic” of inactive lifestyles is still on the rise [1,2]. Sedentary behavior is generally defined as any waking behavior with an energy expenditure ≤ 1.5 METs in a sitting or reclining posture, whereas physical inactivity is characterized by the lack of sufficient moderate- to vigorous-intensity physical activity [3]. Sedentary behavior can therefore be seen as the lowest edge of the physical activity spectrum. An inactive lifestyle is associated with many adverse health effects including increased risk of coronary heart diseases, hypertension, type 2-diabetes, as well as cancer and reduced life expectancy [1,4,5,6,7]. Furthermore, sedentary behavior increases markers associated with inflammation, the risk of obesity, depression, musculoskeletal diseases and osteoporosis risk for women [8,9,10,11,12]. Physical inactivity and sedentary behavior cause not only morbidity and mortality, but also create a major economic burden, especially in high-income countries [13].

A well-known factor associated with physical inactivity is urbanization, which is rapidly increasing worldwide. In 1970, only 36.6% of the world’s population lived in urban settlements. This number had risen to 55.3% in 2018 and is expected to reach 60.4% by 2030 [14]. People living in urban areas are 26% more likely to have a sedentary lifestyle than people living in rural areas [15]. Urbanization leads to a change in lifestyle because work and leisure time activities shift to indoor spaces, thus promoting a more sedentary lifestyle. Especially in high income countries, a significant decrease in physical activity was observed between 2001 (31.6%) and 2016 (36.8%). The same study also indicates that 27.5% of the population worldwide exhibits an insufficient physical activity level [16]. Scientific evidence is growing that physical activity could eliminate or reduce the negative health effects of sedentary behavior, as it reduces the association of sitting time with all-cause mortality and cardiovascular disease mortality [17]. Furthermore, regular physical activity improves immunosurveillance and immunocompetence, induces an anti-inflammatory effect, thus decreasing the risk of the development of non-communicable diseases, acute infection and even has a neuroprotective effect [18]. Exercise may also influence the body’s reward system by altering neurotransmitter levels. 

Growing cities displace nature and, as a consequence, people living in urban regions have restricted access to nature [19], which could be in turn a factor in the vicious cycle of physical inactivity [15]. People who visit local green spaces once a week are four times likelier to reach the recommended amount of physical activity than people who have no access to nature nearby [20]. There is growing evidence that exposure to natural spaces (e.g., forests, blue and green spaces) has a wide range of positive health effects [21,22]. Three main domains of positive health effects through exposure to nature have been identified: (a) reduced exposure to air pollution, heat and noise, (b) restoring capacities, e.g., recovering from stress and (c) building capacities, e.g., encouraging physical activity [23,24]. Green exercise combines the synergistic effects of physical exercise and direct exposure to nature. Several studies found superior health effects of green exercise in comparison to indoor exercise, including better quality of life and mood, reduction of stress, improved cardiovascular health, greater enjoyment and less negative feelings like frustration and even a greater intent to repeat the exercise [25,26,27,28]. However, a recent systematic review could not provide enough high-quality evidence to support the superior health effects of green exercise, and shows the need for further research [29]. Nevertheless, green exercise has one clear advantage over gym-based exercises as there are no restrictions to opening hours and no membership fees for exercising outdoors. 

One popular green exercise activity is mountain hiking. Every year millions of people of all ages spend their holidays in the alpine regions and undertake hiking tours [30]. Mountain hiking can be described as a long-lasting activity under moderate intensity [31]. Although rest and recreation are still the main motives for vacation, physical activity during holidays has increased since the end of the 1990s. Hiking especially is gaining more and more popularity and represents an important travel motive [32]. Looking at the recent fitness trends worldwide and in Europe, personal training is the most popular [33,34]. From this trend, it may be concluded that personal interaction and motivation are critical factors in promoting physical activities. Innovative health coaching approaches combining physical activity and coaching elements could be one way to counteract the global inactivity trend.

Health coaching is a valid method of health education and health promotion within a coaching context, to improve well-being and achieve health-related goals [35]. Besides urbanization, another factor favoring inactivity is the lack of knowledge about the health-promoting effects of physical activity [15]. In this context, health coaching could provide a valuable input, as health coaching can be seen as a combination of health education and behavioral change theory. It includes the following patient-centered methods: (a) identifying and setting personal goals, (b) self-reflection in personal motivational interviews and (c) explaining specific health-related aspects [35,36,37]. Health coaching is a valid method with a full description of the technique for studies and is also causally related to a positive behavioral outcome [37]. In recent years, coaching has grown in popularity in different application areas. Yet, there is also a lack of empirical studies that use control group designs to evaluate the effects of coaching programs [38]. This method especially motivates adults to change their lifestyle, as it supports them in managing their personal goals [39]. It leads to an improvement of physical and mental health and may enhance the quality of life [40]. Most studies using health coaching concern chronic diseases such as diabetes type 2 [39,41]. So, there is a lack of research on the impact of coaching in people with predominantly sedentary lifestyles and the influence on health parameters, especially in terms of gender and age [42].

From the aspect that sedentary behavior at work is not sufficiently compensated during leisure time [43], innovative approaches to promote the enjoyment of physical activity and thus encourage adherence to an active lifestyle supporting a healthy lifespan must be developed. Health coaching in combination with supervised green exercise could initiate long-lasting lifestyle changes. The aim of the presented HICO study was to investigate the effects of a 7-day intervention with green exercise and health coaching on cardiorespiratory fitness and quality of life of sedentary couples. To determine improvements in cardiorespiratory fitness, aerobic capacity and spirometry parameters were measured. For mental health enhancement, questionnaires EQ-5D-5L and SF-36 were provided. Hence, the following hypotheses are approached:

**Hypothesis** **1** **(H1).** *The combination of hiking and coaching improves the cardiorespiratory fitness after 80 days more sustainably than hiking without coaching.*

**Hypothesis** **2** **(H2).** *The combination of hiking and coaching improves the cardiorespiratory fitness after seven days more than hiking without coaching.*

**Hypothesis** **3** **(H3).** *The combination of hiking and coaching improves the quality of life more than hiking without coaching.*

## 2. Materials and Methods

### 2.1. Study Design and Settings

We performed a randomized, controlled trial (HICO Study, https://doi.org/10.1186/ISRCTN25562081 (accessed on 2 December 2021)) to investigate the combined effects of coaching and moderate mountain hiking on the cardiorespiratory fitness of couples with a sedentary lifestyle. In the HICO Study, two intervention groups (hiking and coaching) and one non-intervention control group were included. Only the two intervention groups were finally analyzed due to a high dropout rate and recruitment problems in the control group. Therefore, this work is focused on the comparison of the hiking and coaching group. The allocation ratio for all groups was set at an equal sample size. The hiking group participated in a 7-day mountain hiking program and the coaching group additionally received several coaching sessions with a psychologist. The study protocol was approved by the Ethics Committee of Salzburg (415-E/1488/2-2012) and the study was conducted in Pinzgau (Salzburg Land, Austria) between June and September 2012. Follow-up examinations took place in Salzburg (Austria) between October and December 2012.

### 2.2. Participants

Eligible participants were couples with a sedentary lifestyle. Participants were recruited all over Austria and Germany through advertisements in newspapers and communication via webpage (http://gesund-umdenken.com/was-wir-bieten/klinische-studie/index.html) between May and June 2012. Written informed consent was obtained from all participants. Inclusion criteria were age 22–54 years and a sedentary lifestyle, which means a maximum energy expenditure of 1.0–1.5 metabolic equivalents per day and the physical ability to participate in moderate hiking tours. The questionnaire “Assessment of the Physical Activity Level with two Questions” by Johansson and Westerterp [44] was used as a measure of a sedentary lifestyle. As no official German translation exists, the questionnaire was translated by the authors themselves. Only people scoring ≤ 1.6 were included. Exclusion criteria were as follows: non-sedentary lifestyle (Score > 1.6), cardiovascular diseases, severe hypertension (≥level 3), antihypertensive medication, pulmonary dysfunction, uncontrolled metabolic diseases (e.g., diabetes), malignant neoplastic diseases, orthopedic diseases, acute pain, active infectious diseases and pregnancy. Exclusion criteria were screened during the recruitment process using a short survey. 

### 2.3. Intervention

The study was carried out as part of a 7-day vacation in four regions in Pinzgau (Salzburg Land). The hiking and coaching group participated in an identical mountain hiking exercise program. All participants completed five hiking tours between Sunday and Friday (no hiking on Wednesday) with a daily difference in altitude of at least 600 m. The hiking tours were carried out in comparable mountain massifs, including the High Tauern, Northern Limestone Alps, Berchtesgadener Alps and Kitzbühel Alps. Regarding the technical classification of the trails, mainly “blue” and short stages “red” paths were completed during the hiking; these mean easy and moderately difficult, sometimes also narrow and steep trails, which have no or hardly any areas with a risk of falling. In addition, the coaching group received three individual psychological coaching sessions with a psychologist owning a university diploma in health psychology. All coaching sessions were performed in a quiet and pleasant ambiance at the hotels where all participants stayed overnight. Each coaching session lasted approximately 1.5 h. A follow-up visit was scheduled 80 days after the first intervention. The coaching group received another individual psychological coaching session at the follow-up meeting. All medical examinations were performed by members of the Institute of Ecomedicine and of the Institute of Physiology and Pathophysiology from the Paracelsus Medical University of Salzburg respectively. 

### 2.4. Data Collection and Outcomes

**Figure 1:**
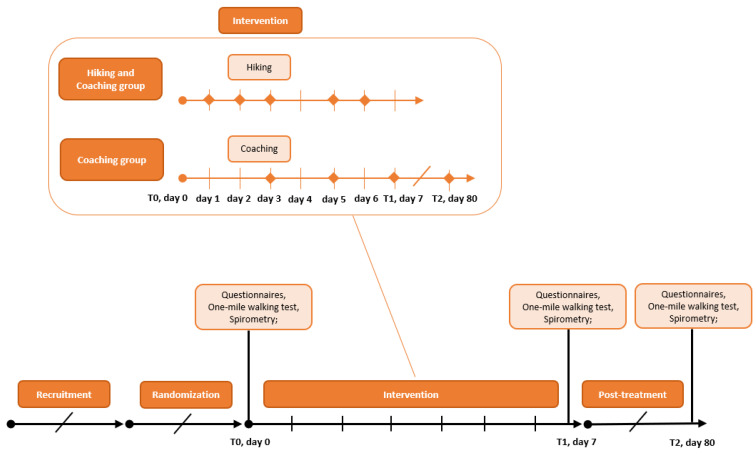
Study schedule for the hiking and the coaching group.

Data were anonymized using four-digit-IDs. The overall trial start was on 1 January 2012. After a preliminary phase, the recruitment of subjects took place from 7 May 2012, followed by randomization. The intervention phase started on 30 June 2012. The study ended with a follow-up phase and data analysis on 1 February 2013. Medical examinations at baseline (T0; day 0) and after the intervention phase (T1; day 7) were performed in mobile lab setups in the participants’ accommodations. Follow-up examinations (day 80; T2) were completed at the Paracelsus Medical University in Salzburg, Austria. Questionnaires were handed out for completion at baseline (T0, day 0), after the intervention phase (T1, day 7) and at the follow-up meeting (T2, day 80). An overview is given in Figure 1. The assessments were conducted, with a warm-up before, outdoors in the summertime at the same time of the day.

#### 2.4.1. One-Mile Walking Test

$V_{O_{2}max}$ [mL kg^−1^ min^−1^] describes the maximum rate of oxygen consumption during exercise. The 1-mile walking test is a validated and economical method to estimate the $V_{O_{2}max}$ indirectly [45]. In preparation for the 1-mile walking test data was collected on weight, age and gender of every person. The values of $V_{O_{2}max}$ are different for gender and age. Men have a higher maximum rate of oxygen consumption than women with a comparable fitness level [46,47]. The participants were asked to walk 1 mile (1.6 km) as fast as possible. At the end of the walk heart rate and oxygen saturation were measured. The estimated $V_{O_{2}max}$ was calculated using the equation by Kline et al. [45]: 6.9652 + (0.0091 × WT) − (0.0257 × AGE) + (0.5955 × SEX) − (0.220 × T1) − (0.0115 × HR); WT = weight in pounds; AGE = Age in years; SEX = Gender: 0 = female, 1 = male; T1 = time for 1 mile in minutes; HR = heart rate.

#### 2.4.2. Questionnaires for Health-Related Quality of Life

TheEuroQOL-5 Dimension Questionnaire version (EQ-5D-5L) involves five dimensions with five levels each. The answers for all dimensions yield a five-digit number that describes the participant’s health status adapted for each country. EQ-5D-5L also includes a visual analogue scale (VAS) which records the participant’s self-rated health state [48,49]. The 36-Item Short Form Health Survey (SF-36) comprises 36 items which can be subsumed into eight concepts: physical functioning, bodily pain, role limitations due to physical health problems, role limitations due to personal or emotional problems, emotional well-being, social functioning, energy/fatigue, and general health perceptions. The scores of each concept range from 0 to 100. The lower the score in a concept, the more limited the participant is [50].

#### 2.4.3. Spirometry

A forced expiratory maneuver was performed according to the manufacturer’s (EasyOne, ndd Medical Technologies, Zurich, Switzerland) and ATS/ERS guidelines [51]. The following parameters were analyzed: forced vital capacity (FVC (%)), forced expiratory volume in 1 s (FEV_1_ (%)) and peak expiratory flow (PEF (%)).

#### 2.4.4. Coaching

The coaching group received three single coaching sessions of 90 min at days 3, 5, 7 and 80. The coaching sessions were standardized and performed by four certified coaches (psychologists, University of Salzburg) with a university diploma in coaching. 

Due to the short intervention time, participants could not receive more coaching sessions as in the usual psychological coaching process. Based on the grow model [52], participants were supported in their goal settings, in checking the actual-/desired condition (reality), in choosing the best and appropriate options, and in beginning the first concrete action steps (will power, what, when, who). 

Session 1: The first session aimed to create an optimal basis for coaching by focusing on the coach-client relationship and creating an acceptance of the method. Each client had the opportunity to set three individual, relevant goals on the subject of health. These goals were roughly classified in the areas of sport, nutrition and stress. The goals were then accurately reflected and operationalized to reduce their abstraction and to increase target specificity and clarity. The final part of the first session was the discussion of the test results of the burnout screening scales [53]. 

Session 2: In the second session, the results of two stress-related questionnaires were discussed. With the help of the trier inventory for chronic stress [54] and the stress processing questionnaire [55] specific stressors of the participants were identified. These results were discussed in detail in the further course of the session in coordination with the client’s goals and roughly analyzed for strengths, weaknesses, opportunities, threats in the sense of the SWOT model.

Session 3: In the third session, the main goal of the client was concretized by intensively discussing the first steps to be taken for the time after the coaching. Clear, measurable and verifiable goals and sub-goals were set by checking them for specificity, measurability, acceptance, feasibility and scheduling on the basis of the SMART criteria. During the preparation of the implementation plan, milestones were also created, other people from the client’s environment were integrated (for example, as initiates in the plans, as feedback providers or as controllers) and a relapse prophylaxis was set up in the event of failure.

#### 2.4.5. Randomization and Sample Size

Randomization was performed with the “Random Allocation Software” (Isfahan, Iran) program with a block randomization protocol [56]. Recruitment of eligible participants, randomization and assignment to treatments were performed by Arnulf Hartl. No a priori sample size calculation was performed.

#### 2.4.6. Statistical Analysis

In an intention-to-treat analysis, all statistical analyses were entered into the R-GNU software environment (General Public License, R Foundation for Statistical Computing, Vienna, Austria, Version 4.0.2). Variables reported in tables were presented as means and their standard deviation, as far as not stated otherwise. Missing values were replaced by two methods: LOCF (last outcome/observation carried forward) if data was missing on day 7 or day 80 by random and NOCB (next outcome/observation carried backward) for missing data on day 0. For all tests, a significance level of 5% probability was set. As the data were not normally distributed, longitudinal data analysis was performed with the nparLD-package [57]. This package offers ANOVA-type statistics for nonparametric longitudinal data analysis. Within the F1-LD-F1-model from the nparLD-package, group (hiking, coaching) was defined as whole-plot-factor and time (T0, T1 and T2) as sub-plot-factor. The F1-LD-F1 model provides an ANOVA-type statistic for group, time and the interaction of group and time (group × time). In case of significant main effects for time or treatment, post hoc tests were applied for a comparison of T0 and T1, respectively, T0 and T2 with another F1-LD-F1-model. Post hoc tests were amended for multiple testing by the Bonferroni–Holm method. 

Next to the ANOVA-type statistic, the F1-LD-F1 model offers relative treatment effects (RTE) as a unitless measure of effect size. The RTE reaches values between 0 and 1 and can be interpreted as follows: An RTE of 0.25 for a certain subgroup means that the probability of a randomly chosen person from this subgroup to score higher than a randomly chosen person from the entire dataset is estimated to be 25%. On the other hand, the probability that a randomly chosen person from this subgroup scores lower than a randomly chosen person from the entire dataset is estimated to be 75%. An RTE equal to 0.50 means no tendency for a higher or lower score in any subgroup.

#### 2.4.7. Sample Size Simulation

In addition, we performed a post hoc sample size calculation with the primary outcome aerobic capacity. In order to meet the requirements of modern statistical approaches, the sample size calculation was performed based on a bootstrap simulation for F1-LD-F1 models and ANOVA [58]. Within the bootstrap simulation, the group size of *n* = 20 to *n* = 70 was varied by steps of ten for each group with random values from the corresponding group (initial seed was set at 1). The statistical power can be estimated for each group size by the percentage of significant (*p*-value < 0.05) counts.

## 3. Results

### 3.1. Study Participants and Baseline Characteristics

**Figure 2:**
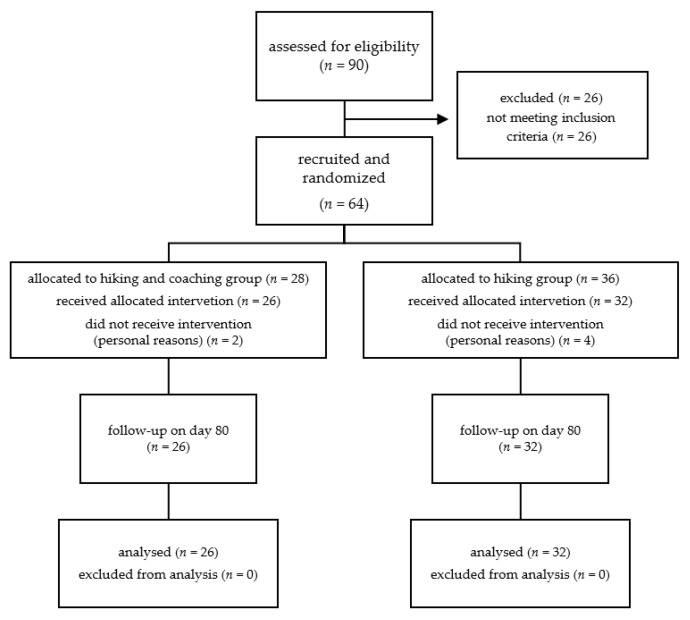
Study flowchart of included and excluded participants.

Out of 90 eligible people, 28 were enrolled for the coaching group and 36 people for the hiking group. In total, 26 people were excluded because of personal reasons or because they did not meet the inclusion criteria. Two participants from the coaching and four from the hiking group declined to participate because of personal reasons. For the statistical analysis, 26 participants of the coaching group and 32 participants of the hiking group were included (Figure 2). All participants tolerated the hiking and coaching program well. No harm or unintended effects were observed. 

Baseline characteristics show no relevant differences between the study groups (Table 1 and Table 2), except for age, $V_{O_{2}max}$ and two variables of the SF-36 questionnaire. The coaching group is significantly older than the hiking group (t (55.1) = −2.92, *p* = 0.01) and the hiking group also shows significantly higher scores for $V_{O_{2}max}$ than the coaching group. The values of physical functioning and bodily pain are significantly higher in the hiking group than in the coaching group (physical functioning: W = 539.5, *p* = 0.03; bodily pain: W = 571.5, *p* = 0.01). Descriptive statistics over all time points are summarized in Table A1 and Table A2 in Appendix A.

### 3.2. One-Mile Walking Test

**Figure 3:**
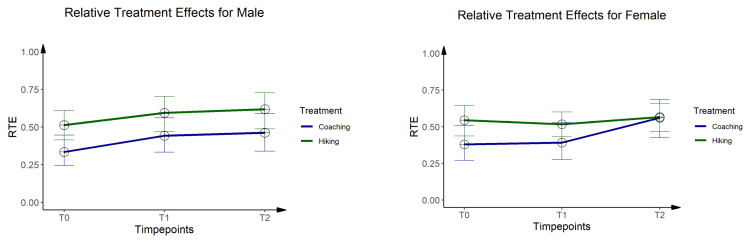
Results from the F1-LD-F1 model for aerobic capacity. Relative treatment effects (RTE) from the F1-LD-F1 models for aerobic capacity for male (**left plot**) and female (**right plot**) participants.

For $V_{O_{2}max}$ of the 1-mile walking the F1-LD-F1-model provided a significant main effect for time (Table 3) but post hoc tests did not show any interaction effects at single time points. Because $V_{O_{2}max}$ depends on gender, women and men were analyzed separately by the F1-LD-F1 models. For men, a significant time effect was found but post hoc tests did not yield any significant effects. The RTEs indicate a parallel development of both groups, whereas the coaching group is generally characterized by lower levels for $V_{O_{2}max}$ (Table 3, Figure 3). For women, a significant main effect was also found for time, but post hoc tests revealed a significant interaction effect on day 80. The RTEs indicate a greater improvement of the coaching group between day 7 and day 80 (Table 3, Figure 3). 

### 3.3. EQ-5D-5L

The F1-LD-F1-model for the visual analogue scale of EQ-5D-5L revealed a significant main effect for time but post hoc tests did not show any interaction effects at the single time points. Both the hiking (83.4 ± 9.71 vs. 86.88 ± 9.31%) and the coaching group (80.38 ± 15.09 vs. 87.31 ± 7.78%) rated their health status better. The analysis of the EQ-5D-5L index showed no significant main effects for treatment, time, or interaction (Table 4).

### 3.4. SF-36

The F1-LD-F1-model for sum scores of the SF-36 questionnaire revealed a significant effect for time in general health, role emotional, physical dimension, mental dimension, and total score, indicating an increase in both groups. Post hoc tests did not show any interaction effects at the single time points. Effects in treatment were significant in physical functioning and bodily pain. For bodily pain, RTEs show higher improvement of the coaching group with time (Table 4).

### 3.5. Spirometry

No significant effects were found for any spirometry parameter (Table 3). The slightly above-average level at baseline of the forced vital capacity (hiking: 109.97 ± 15.72%; coaching: 109.00 ± 15.00%), forced expiratory volume after 1 s (hiking: 105.09 ± 14.32%; coaching: 103.46 ± 13.04%) and peak expiratory flow (hiking: 101.17 ± 17.21%; coaching: 110.84 ± 21.56%) do not change in any group throughout the intervention and post-treatment phase.

### 3.6. Sample Size Simulation

**Figure 4:**
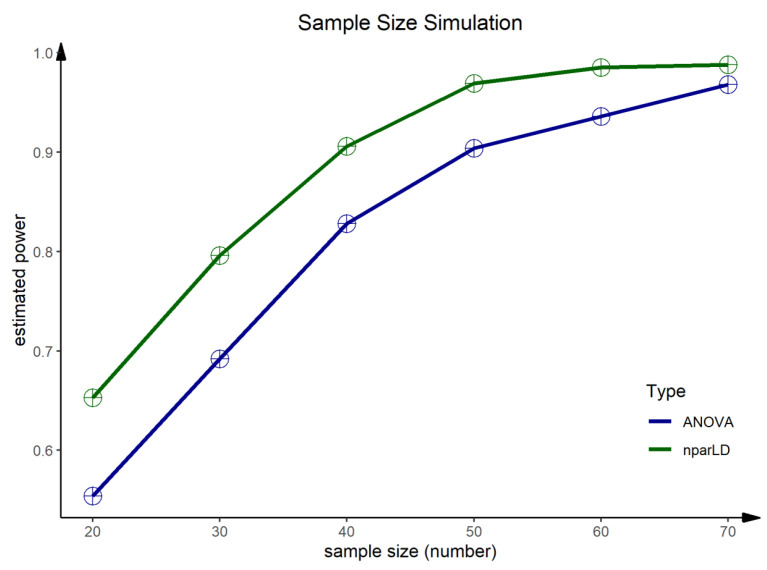
Sample size simulation for $V{\left(O_{2}\right)}_{max}$ for F1-LD-F1 models and ANOVA. Post hoc sample size simulation based on aerobic capacity for F1-LD-F1 models and ANOVA with group, time and group × time interaction effects. For a sample size of *n* ≥ 60 per group, an estimated power of 1 − β = 0.94 can be expected.

The post hoc sample size simulation yielded high differences between the two statistical models (F1-LD-F1 and ANOVA) for sample sizes ≤ 50. (Figure 4, Table A3). The F1-LD-F1 model reaches with *n* = 50 already an acceptable power of 1 − β = 0.97 whereas the estimated power for the ANOVA lies by 1 − β = 0.90. Similar results for both statistical models are evident from a sample size ≥ 60 per group. Hence, in sample sizes *n* ≥ 60 per group an acceptable estimated power of 1 − β ≥ 0.94 can be expected for both statistical models.

## 4. Discussion

A wide range of adverse health effects is associated with sedentary behavior and physical inactivity. Urbanization and lifestyle changes promote an inactive lifestyle, leading to a global increase in chronic diseases [17,59,60]. Therefore, new concepts are urgently needed to bring people back to an active and healthy lifestyle. Health coaching together with moderate mountain hiking as a sport that needs little equipment and personal skills could be instrumentalized to induce a more active lifestyle in the working population. The aim of the presented randomized, controlled trial is to examine the effects of moderate green exercise in form of mountain hiking and health coaching and on the cardiorespiratory fitness and quality of life of couples with a sedentary lifestyle. 

A valid parameter to evaluate cardiorespiratory fitness is spirometry. Within this population of sedentary couples, no significant changes were found for any spirometry parameter, neither as a short-term effect nor a long-term effect. Both groups start with a good lung function and keep this level throughout the intervention and post-treatment phases. As the baseline levels are already above average and the intervention duration is rather short, no relevant changes can be expected either way. Another well-established indicator of cardiorespiratory fitness is aerobic capacity. The direct measurement of aerobic capacity is very time and cost-intensive because of the need for trained staff and technical equipment [61]. For this reason, we performed a less expensive but well-validated method instead: the one-mile walking test. In this submaximal exercise test, the participants are asked to walk one mile as fast as possible [45]. The estimated aerobic capacity was analyzed separately for men and women. Although a significant baseline difference for aerobic capacity was found between the intervention groups, the statistical analysis revealed a significant time effect. The hiking group (9.89 ± 6.35 L/kg × min) starts with a higher aerobic capacity in comparison to the coaching group (44.04 ± 8.94 L/kg × min). During the 7-day intervention, the aerobic capacity is improved in both groups. However, the relative treatment effects indicate a stronger increase in the hiking group, which could be explained by the lower baseline values. Within the female subgroup, a significant time effect was detected, indicating a slight decrease of aerobic capacity in the hiking group during the 7-day intervention, followed by an increase during the post-treatment phase. In contrast, the aerobic capacity of the female participants of the coaching groups improves their aerobic capacity already during the intervention period and shows a clear increase during the post-treatment phase. This different development in the female subgroup is reflected by a significant interaction effect at day 80 (treat × time *p* = 0.03). Although the changes in aerobic capacity occur in a minimal amount, it reveals a possible gender effect: females seem to be receptive to the health coaching approach. 

Looking at *Hypothesis 1—The combination of hiking and coaching improves the cardiorespiratory fitness more sustainably than hiking without coaching* this gender aspect must be considered. Women seem to have a better relation to their feelings and impulses and also tend to attribute their “wrong” behavior to internal causes due to a lack of knowledge and skills [62]. Furthermore, women accept the activities and health recommendations of the coach more than men do, as Linning et al. [63] show in their study on the promotion of fitness and health in employees. Thus, it is not unexpected that women rate their personal coaching process outcome more positively than men. Women seem to be more able to establish good working relations with the coach which also had a positive impact on the evaluation of coaching effectiveness [64]. Other studies also found significant differences in gender because of the interpersonal variation in how people participate in and progress through a health coaching program [65]. Apart from this gender-based coaching effect, further studies should also include the neurological aspects of (green) as Mason et al. [66] show that a diet and exercise intervention with physical activity training can reduce reward-driven eating and, consequently, promote weight loss.

*Within this study population of sedentary couples, no evidence was found to support Hypothesis 2—Combination of hiking and coaching improves cardiorespiratory fitness more than hiking without coaching.* Although the aerobic capacity improves in the male subgroup during the 7-day intervention no superior effects were found for the coaching group. Furthermore, the changes in aerobic capacity are rather small. The baseline levels of spirometry are above average, and the aerobic capacity is also in a normal range. This leaves little space for improvements. However, the questionnaire by Johannsson and Westerterp [45] should be critically evaluated as an inclusion criterion. In addition, no official German translation exists and the translation by the authors themselves may create a bias.

Besides cardiorespiratory fitness, health-related quality of life is an important patient-centered outcome. The SF-36 and the EQ-5D-5L were used for the measurement of the quality of life. The EQ-5D-5L index clearly shows that both groups rate their health status at baseline as already very good. Considering that the maximum score in the EQ-5D-5L index is 1, the average score of both groups of 0.97 leaves almost no room for improvement. However, a significant time effect (time *p* = 0.01) can be observed for the visual analogue scale, indicating a comparable improvement in both groups. The SF-36 questionnaire revealed significant changes over time without any relevant group or interaction effects. Significant time effects were observed for General Health (*p* = 0.01), Role Emotional (*p* = 0.01), Physical Dimension (*p* = 0.04), Mental Dimension (*p* = 0.03) and Total Score (*p* = 0.03), all indicating comparable improvements in both groups during the 7-day intervention. For the Physical Functioning and Physical Pain subscales, there is a significant group effect, which can be attributed to significant differences between the groups at baseline. For health-related quality of life, no indicators were found to support *Hypothesis 3—The combination of hiking and coaching improves the quality of life more than hiking without coaching.* Slight improvements in health-related quality of life can be observed in both groups, without any superior effect of coaching. 

Further research is needed to evaluate the effects of health coaching on improving cardiorespiratory fitness, as our results are limited to a highly functioning sedentary population. Furthermore, the results need to be discussed in the context of the small sample size. As mentioned in the methodology, only two intervention groups were evaluated due to a high dropout rate and recruitment problems in the control group. However, since we have two randomly assigned intervention groups, the study design of a randomized, controlled trial remains. To keep the sample size as high as possible, missing values were reconstructed by the Last Observation Carried Forward Method (LOCF) and the Next Observation Carried Backward Method (NOCB), respectively. Hence, baseline values on day 0 and values on day 7 and day 80 were reconstructed, which might lead to biases in both the short-term and long-term effects due to the small sample size. Another bias within this study is the coaching itself—not only sympathy but also age and gender of the coaches could influence the impact of coaching and the effect of achieving personal goals [57]. However, the presented data shows the feasibility of such approaches. Furthermore, we performed a post hoc sample size simulation to provide a data-based sample size estimation for further studies. The sample size simulation was performed for aerobic capacity with both nonparametric (F1-LD-F1) and parametric models (ANOVA). A sample size of *n* ≥ 60 people should be reached for such study designs to obtain an estimated power of 1 − β ≥ 0.94. 

## 5. Conclusions

Regular exercise in nature can help reduce the risk of cardiovascular diseases, which appear to be a common health problem with a sedentary lifestyle. Mountain hiking and mountain hiking in combination with health coaching are capable of inducing improvements in health-related quality of life and cardiorespiratory fitness. No superior effects of health coaching were found. In further studies, a sample size of *n* ≥ 60 must be achieved in order to gain an acceptable statistical power. 

## Figures and Tables

**Table 1:** Baseline characteristics of the study population.

	Hiking Group (*n* = 32)		Coaching Group (*n* = 26)		Baseline Test	
	Mean ± SD	Median ± IQR	Mean ± SD	Median ± IQR	*p*-Value	Test
Gender	male *n* = 15	female *n* = 17	male *n* = 13	female *n* = 13	1.00	χ² Test
Smoking status	smoker *n* = 5	no-smoker *n* = 27	smoker *n* = 7	no-smoker *n* = 19	0.47	χ² Test
Age (years)	36.59 ± 8.53	34.5 ± 12.75	42.19 ± 6.04	42.50 ± 6.50	0.01 *	T-Test
Height (m)	175.28 ± 9.07	173.5 ± 12.35	173.35 ± 9.01	172.00 ± 14.75	0.42	T-Test
Weight (kg)	75.92 ± 15.87	73.75 ± 15.63	76.31 ± 15.95	71.45 ± 11.85	0.84	U-Test
BMI (kg/m²)	24.63 ± 4.26	24.59 ± 5.09	25.28 ± 4.09	24.63 ± 3.64	0.54	U-Test
FEV_1_ (%)	105.09 ± 14.32	104.26 ± 14.44	103.46 ± 13.04	102.85 ± 12.3	0.65	T-Test
FVC (%)	109.97 ± 15.72	108.84 ± 13.2	109.00 ± 15.00	109.00 ± 11.00	0.77	T-Test
PEF (%)	101.17 ± 17.21	99.68 ± 20.73	110.84 ± 21.56	109.25 ± 30.28	0.07	T-Test
*VO_2max_* (mL/min/kg)	47.83 ± 7.97	49.12 ± 7.26	43.16 ± 7.20	43.34 ± 9.56	0.02 *	T-Test
Male: *VO_2max_*	49.89 ± 6.35	50.79 ± 4.39	44.04 ± 8.94	48.78 ± 9.93	0.06	T-Test
Female: *VO_2max_*	46.01 ± 8.96	45.80 ± 10.71	42.29 ± 5.15	42.77 ± 4.37	0.16	T-Test

IQR: interquartile range; SD: standard deviation; χ² Test: Chi-Square-Test; T-Test: Student’s T-test; U-Test: Mann–Whitney U-test; BMI: body mass index; FEV_1_: forced expiratory flow after 1 s; FVC forced vital capacity; *V_O2max_*: aerobic capacity; * *p* < 0.05.

**Table 2:** Baseline characteristics of questionnaires.

	Hiking Group (*n* = 32)		Coaching Group (*n* = 26)		Baseline Test	
	Mean ± SD	Median ± IQR	Mean ± SD	Median ± IQR	*p*-Value	Test
**EQ-5D-5L**						
-VAS	83.44 ± 9.71	90.00 ± 10.00	80.38 ± 15.09	80.00 ± 20.00	0.55	U-Test
-Score Index	0.97 ± 0.05	1.00 ± 0.08	0.97 ± 0.04	1.00 ± 0.09	0.93	U-Test
**SF-36**						
-Physical Functioning	97.66 ± 4.21	100.00 ± 5.00	95.77 ± 4.17	95.00 ± 5.00	0.03	U-Test
-Role—Physical	92.97 ± 18.22	100.00 ± 0.00	97.12 ± 10.79	100.00 ± 0.00	0.35	U-Test
-Bodily Pain	86.38 ± 18.13	100.00 ± 18.50	73.69 ± 21.40	82.00 ± 22.00	0.01 *	U-Test
-General Health	81.09 ± 11.92	79.50 ± 18.50	76.81 ± 11.09	77.00 ± 20.00	0.23	U-Test
-Vitality	62.66 ± 15.40	62.50 ± 21.25	64.23 ± 12.86	65.00 ± 10.00	0.67	T-Test
-Social Functioning	88.28 ± 14.53	100.00 ± 25.00	85.58 ± 22.83	100.00 ± 25.00	0.83	U-Test
-Role-Emotional	89.58 ± 19.74	100.00 ± 8.33	89.74 ± 24.53	100.00 ± 0.00	0.68	U-Test
-Mental Health	76.62 ± 14.59	80.00 ± 16.00	77.08 ± 10.34	78.00 ± 14.00	0.76	U-Test
-Physical Dimension	84.15 ± 8.95	84.90 ± 8.15	81.52 ± 8.76	83.70 ± 9.15	0.18	U-Test
-Mental Dimension	79.65 ± 11.27	81.52 ± 13.55	78.69 ± 13.04	82.50 ± 16.12	0.96	U-Test
-Total Score	84.40 ± 8.92	86.62 ± 8.21	82.50 ± 10.32	85.94 ± 15.53	0.58	U-Test

IQR: interquartile range; SD: standard deviation; U-Test: Mann–Whitney U-test; EQ-5D-5L: EuroQOL-5 Dimension Questionnaire; VAS: visual analogue scale; SF-36: Short Form-36; * *p* < 0.05.

**Table 3:** Results from the F1-LD-F1 model for aerobic capacity and spirometry.

Parameter	F1-LD-F1 Model				Relative Treatment Effects (RTE)					
		F (df)	*p*-Value		Time		Coaching		Hiking	
**1-mile walking test**										
$V_{O_{2}max}$							Coaching	0.43	Hiking	0.55
	Treat	3.10 (1.00, ∞)	0.08	n.s.	T0	0.45	Co × T0	0.36	Hi × T0	0.54
	Time	6.47 (1.93, ∞)	0.00	***	T1	0.49	Co × T1	0.43	Hi × T1	0.54
	Treat × Time	2.58 (1.93, ∞)	0.08	n.s.	T2	0.54	Co × T2	0.51	Hi × T2	0.58
$V_{O_{2}max}$ male							Coaching	0.41	Hiking	0.58
	Treat	3.00 (1.00, ∞)	0.08	n.s.	T0	0.42	Co × T0	0.33	Hi × T0	0.51
	Time	3.83 (1.50, ∞)	0.03	*	T1	0.52	Co × T1	0.44	Hi × T1	0.59
	Treat × Time	0.05 (1.50, ∞)	0.91	n.s.	T2	0.54	Co × T2	0.46	Hi × T2	0.62
$V_{O_{2}max}$ female							Coaching	0.44	Hiking	0.54
	Treat	1.11 (1.00, ∞)	0.29	n.s.	T0	0.46	Co × T0	0.38	Hi × T0	0.54
	Time	4.82 (1.87, ∞)	0.01	**	T1	0.45	Co × T1	0.39	Hi × T1	0.52
	Treat × Time	2.26 (1.87, ∞)	0.11	n.s.	T2	0.56	Co × T2	0.56	Hi × T2	0.57
	Time T1	0.02 (1.00, ∞)	0.90	n.s.						
	Time T2	8.65 (1.00, ∞)	0.01	**						
	Treat × T1	0.11 (1.00, ∞)	0.75	n.s.						
	Treat × T2	5.97 (1.00, ∞)	0.03	*						
**Spirometry**										
FVC %							Coaching	0.50	Hiking	0.50
	Treat	0.01 (1.00, ∞)	0.92	n.s.	T0	0.51	Co × T0	0.51	Hi × T0	0.51
	Time	0.77 (1.75, ∞)	0.45	n.s.	T1	0.48	Co × T1	0.47	Hi × T1	0.50
	Treat × Time	0.37 (1.75, ∞)	0.66	n.s.	T2	0.51	Co × T2	0.51	Hi × T2	0.50
FEV_1_ %							Coaching	0.49	Hiking	0.51
	Treat	0.07 (1.00, ∞)	0.80	n.s.	T0	0.52	Co × T0	0.52	Hi × T0	0.53
	Time	2.82 (2.00, ∞)	0.06	n.s.	T1	0.48	Co × T1	0.47	Hi × T1	0.49
	Treat × Time	0.00 (2.00, ∞)	1.00	n.s.	T2	0.49	Co × T2	0.48	Hi × T2	0.50
PEF %							Coaching	0.57	Hiking	0.44
	Treat	3.28 (1.00, ∞)	0.07	n.s.	T0	0.52	Co × T0	0.60	Hi × T0	0.44
	Time	2.10 (1.88, ∞)	0.13	n.s.	T1	0.52	Co × T1	0.59	Hi × T1	0.46
	Treat × Time	0.77 (1.88, ∞)	0.46	n.s.	T2	0.47	Co × T2	0.52	Hi × T2	0.43

F1-LD-F1 model with time and treatment (hiking or coaching) and the interaction of treatment and time (treat × time); df: degree of freedom; time points T0 = day 0, T1 = day 7, T2 = day 80; Hi: hiking-group, Co: coaching-group; FVC: forced vital capacity; FEV: forced expiratory volume at 1 s; PEF: peak expiratory flow; *** *p* < 0.001; ** *p* < 0.01; * *p* < 0.05; n.s. not significant.

**Table 4:** Results from the F1-LD-F1 model for EQ-5D-5L and SF-36 questionnaires.

Parameter	F1-LD-F1 Model				Relative Treatment Effects (RTE)					
		F (df)	*p*-Value		Time		Coaching		Hiking	
**EQ-5D-5L**										
VAS							Coaching	0.49	Hiking	0.51
	Treat	0.10 (1.00, ∞)	0.76	n.s.	T0	0.46	Co × T0	0.44	Hi × T0	0.48
	Time	5.52 (1.73, ∞)	0.01	**	T1	0.57	Co × T1	0.57	Hi × T1	0.57
	Treat × Time	0.22 (1.73, ∞)	0.77	n.s.	T2	0.47	Co × T2	0.46	Hi × T2	0.48
Score							Coaching	0.48	Hiking	0.52
	Treat	0.48 (1.00, ∞)	0.49	n.s.	T0	0.46	Co × T0	0.46	Hi × T0	0.47
	Time	2.95 (2.00, ∞)	0.05	n.s.	T1	0.5	Co × T1	0.46	Hi × T1	0.54
	Treat × Time	0.84 (2.00, ∞)	0.43	n.s.	T2	0.53	Co × T2	0.51	Hi × T2	0.55
**SF-36**										
Physical Functioning							Coaching	0.43	Hiking	0.56
	Treat	6.27 (1.00, ∞)	0.01	*	T0	0.45	Co × T0	0.38	Hi × T0	0.53
	Time	2.63 (1.98, ∞)	0.07	n.s.	T1	0.49	Co × T1	0.44	Hi × T1	0.54
	Treat × Time	0.24 (1.98, ∞)	0.78	n.s.	T2	0.54	Co × T2	0.47	Hi × T2	0.60
Role—Physical							Coaching	0.50	Hiking	0.50
	Treat	0.00 (1.00, ∞)	0.97	n.s.	T0	0.5	Co × T0	0.52	Hi × T0	0.48
	Time	0.39 (1.80, ∞)	0.65	n.s.	T1	0.49	Co × T1	0.50	Hi × T1	0.48
	Treat × Time	2.15 (1.80, ∞)	0.12	n.s.	T2	0.51	Co × T2	0.48	Hi × T2	0.54
Bodily Pain							Coaching	0.43	Hiking	0.55
	Treat	3.95 (1.00, ∞)	0.05	*	T0	0.47	Co × T0	0.38	Hi × T0	0.57
	Time	0.74 (1.99, ∞)	0.48	n.s.	T1	0.5	Co × T1	0.47	Hi × T1	0.53
	Treat × Time	1.88 (1.99, ∞)	0.15	n.s.	T2	0.51	Co × T2	0.46	Hi × T2	0.57
General Health							Coaching	0.45	Hiking	0.54
	Treat	2.09 (1.00, ∞)	0.15	n.s.	T0	0.42	Co × T0	0.37	Hi × T0	0.37
	Time	6.51 (1.82, ∞)	0.01	**	T1	0.54	Co × T1	0.49	Hi × T1	0.49
	Treat × Time	0.09 (1.82, ∞)	0.90	n.s.	T2	0.53	Co × T2	0.49	Hi × T2	0.49
Vitality							Coaching	0.50	Hiking	0.50
	Treat	0.00 (1.00, ∞)	0.96	n.s.	T0	0.47	Co × T0	0.48	Hi × T0	0.45
	Time	1.49 (1.63, ∞)	0.23	n.s.	T1	0.51	Co × T1	0.49	Hi × T1	0.54
	Treat × Time	0.76 (1.63, ∞)	0.44	n.s.	T2	0.52	Co × T2	0.53	Hi × T2	0.51
Social Functioning							Coaching	0.50	Hiking	0.50
	Treat	0.01 (1.00, ∞)	0.91	n.s.	T0	0.48	Co × T0	0.49	Hi × T0	0.47
	Time	0.69 (1.93, ∞)	0.49	n.s.	T1	0.5	Co × T1	0.49	Hi × T1	0.51
	Treat × Time	0.28 (1.93, ∞)	0.75	n.s.	T2	0.52	Co × T2	0.53	Hi × T2	0.51
Role—Emotional							Coaching	0.49	Hiking	0.50
	Treat	0.06 (1.00, ∞)	0.80	n.s.	T0	0.46	Co × T0	0.47	Hi × T0	0.44
	Time	6.64 (1.88, ∞)	0.01	**	T1	0.5	Co × T1	0.51	Hi × T1	0.50
	Treat × Time	1.65 (1.88, ∞)	0.19	n.s.	T2	0.54	Co × T2	0.51	Hi × T2	0.57
Mental Health							Coaching	0.50	Hiking	0.50
	Treat	0.00 (1.00, ∞)	0.95	n.s.	T0	0.47	Co × T0	0.46	Hi × T0	0.49
	Time	1.27 (1.71, ∞)	0.28	n.s.	T1	0.49	Co × T1	0.50	Hi × T1	0.49
	Treat × Time	0.19 (1.71, ∞)	0.79	n.s.	T2	0.53	Co × T2	0.54	Hi × T2	0.53
Physical Dimension							Coaching	0.45	Hiking	0.54
	Treat	1.66 (1.00, ∞)	0.20	n.s.	T0	0.45	Co × T0	0.40	Hi × T0	0.50
	Time	3.22 (1.93, ∞)	0.04	*	T1	0.51	Co × T1	0.47	Hi × T1	0.55
	Treat × Time	0.06 (1.93, ∞)	0.94	n.s.	T2	0.53	Co × T2	0.49	Hi × T2	0.57
Mental Dimension							Coaching	0.50	Hiking	0.50
	Treat	0.00 (1.00, ∞)	0.94	n.s.	T0	0.45	Co × T0	0.45	Hi × T0	0.45
	Time	3.65 (1.70, ∞)	0.03	*	T1	0.51	Co × T1	0.50	Hi × T1	0.52
	Treat × Time	0.13 (1.70, ∞)	0.84	n.s.	T2	0.54	Co × T2	0.54	Hi × T2	0.53
Total Score							Coaching	0.48	Hiking	0.51
	Treat	0.20 (1.00, ∞)	0.65	n.s.	T0	0.45	Co × T0	0.43	Hi × T0	0.47
	Time	3.73 (1.76, ∞)	0.03	*	T1	0.51	Co × T1	0.49	Hi × T1	0.52
	Treat × Time	0.09 (1.76, ∞)	0.89	n.s.	T2	0.54	Co × T2	0.53	Hi × T2	0.55

F1-LD-F1 model with time and treatment (hiking or coaching) and the interaction of treatment and time (treat × time); df: degree of freedom; time points T0 = day 0, T1 = day 7, T2 = day 80; Hi: hiking-group, Co: coaching-group; VAS: visual analogue scale; ** *p* < 0.01; * *p* < 0.05; n.s. not significant.

## Data Availability

The data presented in this study are available on request from the corresponding author.

## Abbreviations

ANOVA	Analysis of variance
ATS/ERS	American thoracic society/ European respiratory society
EQ-5D-5L	EuroQol health survey with 5 dimensions and 5 levels
FEV1	Forced expiration volume in 1 s
FVC	Forced expiration volume
GE	Green exercise
HICO	Hiking and coaching
HR	Heart rate
ID	Identifier
IQR	Interquartile range
km	Kilometers
LOCF	Last outcome/observation carried forward
MET	Metabolic equivalent
NCDs	Non-communicable diseases
NOCB	Next outcome/observation carried backward
nparLD	Nonparametric longitudinal data analysis
PEF	Peak expiratory flow
RTE	Relative treatment effects
SD	Standard deviation
SF-36	Short form health survey with 36 items
T0	time of measurement/day 0
T1	time of measurement/day 7
T2	time of measurement/day 80
VAS	Visual analogue scale
V(O_2)max_	Maximal oxygen consumption or aerobic capacity
WT	Weight in pounds

## Appendix A

**Table A1:** Characteristics of the study population at day 7.

	Hiking Group (*n* = 32)		Coaching Group (*n* = 26)	
	Mean ± SD	Median ± IQR	Mean ± SD	Median ± IQR
Weight (kg)	76.69 ± 15.64	75.85 ± 15.45	76.86 ± 16.04	71.70 ± 11.60
BMI	24.89 ± 4.24	24.64 ± 4.87	25.46 ± 4.06	24.98 ± 3.57
FEV_1_ (%)	103.07 ± 12.9	101.11 ± 18.25	101.72 ± 12.44	102.62 ± 17.50
FVC (%)	108.90 ± 13.24	109.50 ± 13.83	107.82 ± 12.90	107.71 ± 14.80
PEF (%)	100.92 ± 19.91	103.43 ± 27.39	109.51 ± 18.49	112.86 ± 25.15
$V_{O_{2}max}$	48.29 ± 7.54	47.94 ± 8.98	45.62 ± 5.65	46.54 ± 8.95
$V_{O_{2}max}$ (male)	52.27 ± 7.04	52.04 ± 6.74	48.32 ± 5.81	48.68 ± 6.09
$V_{O_{2}max}$ (female)	44.78 ± 6.24	45.55 ± 5.77	42.92 ± 4.10	42.46 ± 7.15
**EQ-5D-5L**				
-VAS	86.88 ± 9.31	90.00 ± 10.00	87.31 ± 7.78	90.00 ± 10.00
-Score Index	0.98 ± 0.04	1.00 ± 0.00	0.97 ± 0.04	1.00 ± 0.09
**SF-36**				
-Physical Functioning	97.66 ± 5.53	100.00 ± 5.00	96.73 ± 3.73	97.50 ± 5.00
-Role—Physical	93.75 ± 16.80	100.00 ± 0.00	95.19 ± 14.18	100.00 ± 0.00
-Bodily Pain	83.78 ± 20.16	84.00 ± 26.00	81.12 ± 17.30	84.00 ± 26.00
-General Health	85.19 ± 12.39	84.50 ± 20.00	81.58 ± 10.47	82.00 ± 9.50
-Vitality	67.34 ± 15.40	67.50 ± 25.00	64.81 ± 12.53	65.00 ± 10.00
-Social Functioning	90.23 ± 14.10	100.00 ± 25.00	88.94 ± 16.33	100.00 ± 12.50
-Role-Emotional	94.79 ± 14.93	100.00 ± 0.00	93.59 ± 18.90	100.00 ± 0.00
-Mental Health	77.25 ± 12.50	78.00 ± 17.00	78.15 ± 11.82	80.00 ± 16.00
-Physical Dimension	85.54 ± 9.37	86.70 ± 9.15	83.88 ± 7.97	84.20 ± 8.35
-Mental Dimension	82.96 ± 8.44	84.30 ± 10.32	81.41 ± 11.16	84.25 ± 9.83
-Total Score	86.25 ± 7.77	86.73 ± 7.70	85.01 ± 8.81	86.56 ± 9.62

IQR: interquartile range; SD: standard deviation; BMI: body mass index; FEV1: forced expiratory flow after 1 s; FVC: forced vital capacity; *VO_2max_*: aerobic capacity; EQ-5D-5L: EuroQOL-5 Dimension Questionnaire; VAS: visual analogue scale; SF-36: Short Form-36.

**Table A2:** Characteristics of the study population at day 80.

	Hiking Group (*n* = 32)		Coaching Group (*n* = 26)	
	Mean ± SD	Median ± IQR	Mean ± SD	Median ± IQR
Weight (kg)	76.36 ± 15.90	73.95 ± 16.05	75.82 ± 15.43	70.65 ± 12.40
BMI	24.79 ± 4.37	24.44 ± 5.13	25.12 ± 3.91	24.98 ± 3.15
FEV_1_ (%)	103.50 ± 12.77	101.58 ± 17.14	102.03 ± 11.45	103.93 ± 9.86
FVC (%)	109.22 ± 13.86	109.84 ± 14.36	109.54 ± 12.49	109.37 ± 15.01
PEF (%)	100.83 ± 16.40	97.57 ± 22.26	105.36 ± 14.32	106.44 ± 21.59
$V_{O_{2}max}$	48.87 ± 7.75	49.47 ± 9.33	48.06 ± 7.56	47.03 ± 7.31
$V_{O_{2}max}$ (male)	51.85 ± 7.26	52.92 ± 8.22	50.31 ± 9.08	49.52 ± 5.70
$V_{O_{2}max}$ (female)	46.24 ± 7.39	45.21 ± 8.85	45.81 ± 5.06	45.83 ± 7.55
**EQ-5D-5L**				
-VAS	83.12 ± 12.30	80.00 ± 10.00	82.69 ± 10.79	80.00 ± 10.00
-Score Index	0.98 ± 0.04	1.00 ± 0.00	0.98 ± 0.04	1.00 ± 0.07
**SF-36**				
-Physical Functioning	98.44 ± 4.66	100.00 ± 0.00	96.92 ± 4.26	100.00 ± 5.00
-Role—Physical	96.88 ± 17.68	100.00 ± 0.00	95.19 ± 12.29	100.00 ± 0.00
-Bodily Pain	86.50 ± 18.35	100.00 ± 26.00	79.12 ± 20.36	84.00 ± 35.00
-General Health	84.44 ± 11.16	87.00 ± 15.75	81.38 ± 12.02	82.00 ± 16.00
-Vitality	65.47 ± 19.97	67.50 ± 36.25	66.15 ± 16.14	70.00 ± 15.00
-Social Functioning	88.28 ± 19.03	100.00 ± 12.50	88.94 ± 19.47	100.00 ± 12.50
-Role-Emotional	100.00 ± 0.00	100.00 ± 0.00	96.15 ± 10.86	100.00 ± 0.00
-Mental Health	77.38 ± 18.36	80.00 ± 14.00	78.77 ± 12.12	84.00 ± 13.00
-Physical Dimension	86.34 ± 9.32	86.90 ± 10.25	83.75 ± 9.42	86.50 ± 12.00
-Mental Dimension	83.11 ± 10.12	84.65 ± 10.45	82.28 ± 10.92	86.20 ± 11.20
-Total Score	87.17 ± 7.73	87.16 ± 8.95	85.33 ± 9.45	88.94 ± 12.83

IQR: interquartile range; SD: standard deviation; BMI: body mass index; FEV1: forced expiratory flow after 1 s; FVC: forced vital capacity; *VO_2max_*: aerobic capacity; EQ-5D-5L: EuroQOL-5 Dimension Questionnaire; VAS: visual analogue scale; SF-36: Short Form-36.

**Table A3:** Sample size simulation for aerobic capacity.

Number	Estimated Power	
	ANOVA	F1-LD-F1
20	0.55	0.65
30	0.69	0.80
40	0.83	0.91
50	0.90	0.97
60	0.94	0.99
70	0.97	0.99

Results from the post hoc sample size simulation for F1-LD-F1 models and ANOVA with group, time and group × time interaction effects.
